# Eliminating or blocking 12/15-lipoxygenase reduces neutrophil recruitment in mouse models of acute lung injury

**DOI:** 10.1186/cc11518

**Published:** 2012-09-13

**Authors:** Jan Rossaint, Jerry L Nadler, Klaus Ley, Alexander Zarbock

**Affiliations:** 1 Department of Anesthesiology, Intensive Care and Pain Medicine, University of Münster, Albert-Schweitzer-Campus 1, Gebäude A1, Münster, 48149, Germany; 2 Department of Internal Medicine, Eastern Virginia Medical School, P.O. Box 1980, Norfolk, VA 23501-1980, USA; 3 Division of Inflammation Biology, La Jolla Institute for Allergy and Immunology, 9420 Athena Circle, La Jolla, CA 92037, USA

**Keywords:** lipoxygenase, acute lung injury, inflammation, leukocyte recruitment

## Abstract

**Introduction:**

Acute lung injury (ALI) is a common disease in critically ill patients with a high morbidity and mortality. 12/15-lipoxygenase (12/15-LO) is an enzyme generating 12-hydroxy-eicosatetraenoic acid (12-HETE) and 15-HETE from arachidonic acid. It has been shown that 12/15-LO is involved in the regulation of vascular permeability during ALI.

**Methods:**

To test whether 12/15-LO participates in leukocyte recruitment into the lung, we investigated the role of 12/15-LO in mouse models of lipopolysaccharide (LPS)-induced pulmonary inflammation and acid-induced ALI, a clinically relevant model of acute lung injury.

**Results:**

The increase in neutrophil recruitment following LPS inhalation was reduced in 12/15-LO-deficient (*Alox15^-/-^*) mice and in wild-type (WT) mice after the blocking of 12/15-LO with a pharmacological inhibitor. Bone marrow chimeras revealed that 12/15-LO in hematopoietic cells regulates neutrophil accumulation in the interstitial and alveolar compartments, whereas the accumulation of neutrophils in the intravascular compartment is regulated by 12/15-LO in non-hematopoietic and hematopoietic cells. Mechanistically, the increased plasma levels of the chemokine CXCL1 in *Alox15^-/- ^*mice led to a reduced response of the neutrophil chemokine receptor CXCR2 to stimulation with CXCL1, which in turn abrogated neutrophil recruitment. *Alox15^-/- ^*mice also showed decreased edema formation, reduced neutrophil recruitment and improved gas exchange in an acid-induced ALI model.

**Conclusions:**

Our findings suggest that 12/15-LO modulates neutrophil recruitment into the lung by regulating chemokine/chemokine receptor homeostasis.

## Introduction

Acute lung injury (ALI) is a severe disease with a high incidence [1]. ALI is associated with high mortality despite improved treatment [1], causes 3.6 million hospital days per year, and thus is a huge burden for the health care system [1]. ALI can occur as a consequence of extrapulmonary causes, including massive transfusion and sepsis, and intrapulmonary causes, such as aspiration of gastric content and pneumonia. During the development of ALI, the alveolar-capillary integrity is compromised, leading to neutrophil infiltration, interstitial and alveolar edema formation, and diminished gas exchange [2]. As yet, there are no specific treatments available for ALI.

Accumulation and recruitment of neutrophils to the lung are key events in the development of ALI [2]. Neutrophil recruitment to the lung occurs in a cascade-like process of activation, intravascular accumulation, and transendothelial and transepithelial migration [3]. Depending on the underlying cause of ALI, different chemokines and adhesion molecules participate in neutrophil recruitment [4]. It has been shown that CXCR2, the chemokine receptor for CXCL1 (keratinocyte-derived chemokine, KC) and CXCL2/3 (macrophage inflammatory protein 2, MIP-2) in the mouse system, is involved in the regulation of vascular permeability and in neutrophil recruitment in different models of ALI [5-7]. Following lipopolysaccharide (LPS) inhalation, CXCR2 on non-hematopoietic and hematopoietic cells each contributes about equally to neutrophil recruitment into the lung [5]. Blocking of CXCR2 by a pharmacological inhibitor or elimination of CXCR2 in both cell types completely abolished neutrophil recruitment into the alveolar space in response to inhaled LPS [5,7]. A recently published study demonstrated that human CXCL8 (interleukin 8; IL-8), a ligand for human CXCR1 and CXCR2 [8] can form complexes with autoantibodies associated with the development and outcome of ALI in patients [9].

Besides chemokines, lipid mediators are critically involved in the development of pulmonary inflammation and play important roles in the pathophysiology of allergen-induced inflammation and lung remodeling in asthma [10,11]. Lipoxygenases (LOX) are a family of enzymes capable of incorporating oxygen into unsaturated fatty acids [12]. Their nomenclature depends upon the position of the carbon double bonds they oxidize counted from the carboxyl terminus of the fatty acid [13]. 15-lipoxygenase is expressed in humans and rabbits (15-LO) [14,15]. "Leukocyte-type" 12-LO, which has some 15-LO activity, is expressed in pigs, rats and mice [16]. A high homology exists between 12/15-LO in mice and 15-LO in humans. Thus, the murine 12/15-LO is most likely the mouse ortholog of human 15-LO [17,18]. The most common substrate for 12/15-LO, arachidonic acid, is released from membrane phospholipids by phospholipase A2 in response to inflammatory stimuli [19]. 12/15-LO metabolizes arachidonic acid. This process yields short-lived, peroxidized products, which are reduced or enzymatically converted to 12-hydroxyeicosatetraenoic acid (12-HETE) as well as hepoxilins, lipoxins and others [20]. 12/15-LO is expressed in a variety of tissues, with the highest expression levels in monocytes and macrophages [21]. Lower expression levels of 12/15-LO are found in endothelial cells [22] and other tissues. 12-HETE has a variety of biological functions. It is a potent, proinflammatory chemoattractant for neutrophils [23]. 12-HETE exerts effects on endothelial cells as it influences cytoskeleton rearrangement [24] and cytokine production [25]. 12-HETE may also induce adhesion molecule expression on endothelial cells [26]. Furthermore, an implication of 12/15-LO in chronic inflammatory processes has been reported [21]. In a previous study, the development of allergic sensitization and airway inflammation has been demonstrated to be diminished in *Alox15^-/- ^*mice [11,27]. However, rabbits made transgenic for human *Alox15 *were protected from atherosclerosis [28] or LTB_4_- or IL-8-induced dermatitis [29], possibly through protective metabolites. Besides 12/15-LO, the lipoxygenase 5-LO has been shown to be involved in inflammatory processes, for example, the ventilator-induced lung injury model [30]. We previously demonstrated that 12-HETE, the product of 12/15-LO, produced by hematopoietic cells plays a key role in the regulation of vascular permeability in LPS-induced pulmonary inflammation and acid-induced ALI through a CXCR2-dependent mechanism [31].

The aim of this study was to investigate the role of 12/15-lipoxygenase on neutrophil recruitment into the lung in murine models of LPS-induced pulmonary inflammation and acid-induced acute lung injury. To reveal the main source of 12/15-LO influencing neutrophil recruitment, chimeric mice were generated by bone marrow transplantation. To reveal the mechanisms by which 12/15-LO modulates neutrophil recruitment, we investigated the role of 12/15-LO on the chemokine/chemokine receptor homeostasis.

## Materials and methods

### Animals

The animals used in this study were 8- to 12-week old C57BL/6 mice (The Jackson Laboratory, Bar Harbor, ME, USA) and 12/15-LO deficient mice (*Alox15^-/-^*) backcrossed to C57BL/6 for at least 10 generations [32]. The mice were kept in a barrier facility under specific pathogen-free conditions. All animal experiments were approved by the Animal Care and Use Committees of the University of Virginia (Charlottesville) and the University of Münster (Germany).

### Murine model of LPS-induced pulmonary inflammation and acid-induced ALI

For the induction of pulmonary inflammation, mice were placed in an enclosed chamber and exposed to nebulized LPS from *Salmonella enteritidis *(500 µg/ml, Sigma-Aldrich, St. Louis, MO, USA) for 30 minutes, as previously described [7]. Control mice were exposed to aerosolized, sterile saline for 30 minutes.

For some experiments, mice were injected with CDC (Cinnamyl-3,4-Dihydroxy-a-Cyanocinnamate, Biomol International, Philadelphia, PA, USA), a pharmacological inhibitor of 12/15-LO. Mice received a single injection (8 mg/kg, intraperitoneal (i.p.)) one hour before LPS application.

Acid-induced ALI was performed as previously described [33]. Briefly, after induction of general anesthesia with ketamine (125 μg/g body weight; Sanofi-Aventis, Paris, France), xylazine (12.5 μg/g body weight; Phoenix Scientific, Inc., St. Joseph, MO, USA), and atropine sulfate (0.025 μg/g body weight; Fujisawa, Tokyo, Japan) mice were placed on a heating pad and the ventral aspect of the neck was incised to gain access to the trachea. A total of 2 µl/g HCl (pH 1.5) was injected intratracheally, followed by a bolus of air (30 µl/g). Tracheotomy was performed and mice were mechanically ventilated with a respirator (MiniVent Type 845, Hugo Sachs Elektronik, March-Hugstetten, Germany) for two hours (tidal volume 10 µl/g; respiration rate 140/minute; fraction of inspiratory oxygen (FiO_2_) 0.21). Control animals were treated in the same way, except for the instillation of sterile saline instead of HCl.

### Neutrophil recruitment into the lung

Twenty-four hours after LPS-inhalation mice were euthanized. Bronchoalveolar lavage (BAL) fluid was collected (5 × 1 ml phosphate-buffered saline). The BAL was centrifuged and the neutrophils in the BAL fluid were counted using Kimura staining.

Intravascular and interstitial neutrophils were distinguished by a flow cytometry-based method as described previously [33]. Briefly, Alexa 633-labeled GR-1 antibody (clone RB6-8C5, staining kit: Invitrogen Corp., Carlsbad, CA, USA) to murine neutrophils was injected intravenously five minutes prior to euthanasia. This procedure labels only intravascular neutrophils as the time period is not sufficient for the antibody to react with neutrophils located in the interstitial or intraalveolar compartment. Thoracotomy was performed, the inferior vena cava was opened and non-adherent neutrophils were removed from the pulmonary vasculature by perfusing the right ventricle with 3 ml of saline with a constant pressure of 25 cmH_2_O. After collection of the BAL, the lungs were removed, minced and digested with an enzyme cocktail (collagenase type XI, hyaluronidase type I-s, DNAse1, Sigma-Aldrich, St. Louis, MO, USA) containing 65 µg unlabelled GR-1-antibody at 37°C for 60 minutes. A cell suspension was made by passing the digested lung tissue through a 70 µm cell strainer (BD Falcon, Bedford, MA, USA). Red blood cells were lysed and the remaining leukocytes were resuspended in PBS and counted. The fraction of neutrophils in the suspension was determined by flow cytometry (FacsCalibur; BD Biosciences, San Jose, CA, USA). Neutrophils were identified by their typical appearance in the forward/sideward scatter (FSC/SSC) and their expression of CD45 (clone 30-F11), 7/4 (clone 7/4, both BD Pharmingen, San Diego, CA, USA), and GR-1 (clone RB6-8C5, purified from hybridoma supernatant). The labelled GR-1-antibody was used to differentiate between intravascular (CD45^+^7/4^+^GR-1^+^) and interstitial (CD45^+^7/4^+^GR-1^-^) neutrophils.

### In vitro chemotaxis assay

The *in vitro *chemotaxis assay was performed as described previously [5]. For the *in vitro *chemotaxis assay, isolated neutrophils were labeled with calcein AM (5 μM; Molecular Probes, Eugene, OR, USA) [34]. A 96-well chemotaxis system (ChemoTx, 3-µm filter pore size; Neuro Probe, Inc., Gaithersburg, MD, USA) was loaded with triplicates of recombinant CXCL1 (1-10,000 ng/ml; PeproTech, Rocky Hill, NJ, USA) or fMLP (n-formyl-methionine-leucine-phenylalanine, 1 to 10,000 ng/ml; Sigma-Aldrich, Taufkirchen, Germany). Neutrophil suspension (2.5 × 10^5^) was placed on top of the filter above each well, and the chamber was incubated for one hour at 37°C [5]. After incubation, non-migrated cells and the filter were removed and fluorescence in the wells was measured with a fluorescence plate reader.

### Generation of 12/15-Lipoxygenase chimeric mice

To investigate the role of 12/15-Lipoxygenase from non- and hematopoietic origin in pulmonary inflammation, we generated chimeric mice as described previously [33]. Destruction of the native bone marrow in male recipient mice was archived by fractional lethal irradiation in two doses of 600 rad each (separated by four hours). WT (C57BL/6) and 12/15-lipoxygenase-deficient mice served as donors and/or recipients. The bone marrow isolation from donor mice was performed under sterile conditions. Approximately 5 × 10^6 ^cells were injected intravenously into irradiated recipient mice. Experiments with chimeric mice were performed six weeks after successful bone marrow transplantation.

### Histology

To investigate changes in tissue morphology following LPS-induced pulmonary inflammation, lungs were removed, embedded in paraffin and cut in 5 µm thick slices. Tissue samples were stained for CXCL1 (anti-murine KC, PeproTech, Rocky Hill, NJ, USA) using the avidin-biotin technique (Vector Laboratories, Burlingame, CA, USA) as described previously [35].

### Surface staining and flow cytometry analysis

We analyzed the surface expression of CXCR2 on peripheral blood leukocytes by incubating whole blood samples with CD45 (clone 30-F11), 7/4 (clone 7/4, both BD Biosciences-Pharmingen, San Diego, CA, USA), GR-1 (clone RB6-8C5), and CXCR2 (clone 242216, R&D Systems, Inc., Minneapolis, MN, USA). After red blood cell lysis with 1.5 M NH_4_Cl, flow cytometry was performed to quantitatively determine surface expression of CXCR2 (FacsCalibur, BD Biosciences, Franklin Lakes, NJ, USA).

### CXCL1/2 measurement

CXCL1 and CXCL2 levels in the plasma with and without LPS challenge were measured in triplicates using ELISA kits according to the manufacturer's protocol (R&D Systems, Minneapolis, MN, USA). In order to measure CXCL1 and CXCL2 in the lung tissue, lungs were mechanically minced and incubated with extraction buffer containing 0.1% Triton X-100, 10 mM EDTA, 100 mM e-aminocaproic acid, 0.01 mM Pepstatin, 0.005 mM Leupeptin and 50 mM Tris at 4°C for 18 hours. Samples were centrifuged and the CXCL1 and CXCL2 concentration in the supernatant was quantified using the ELISA kits as described above.

### Statistics

Statistical analysis was performed with SPSS (version 9.0; SPSS, Inc., Chicago, IL, USA). Statistical tests included one-way analysis of variance, Student-Newman-Keuls test, and t-test where appropriate. All data are presented as mean ± SEM, *P *< 0.05 was considered significant.

## Results

### 12/15-lipoxygenase is involved in LPS-induced pulmonary inflammation

**Figure 1:**
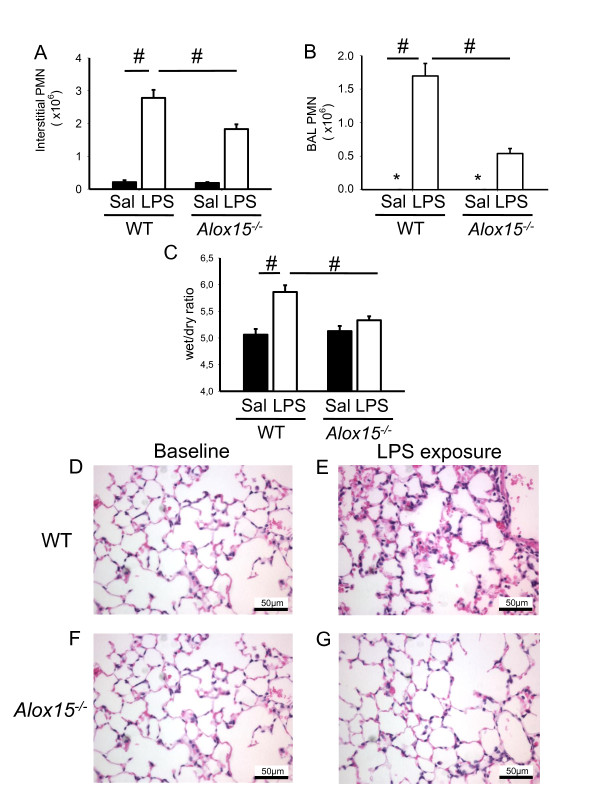
**12/15-lipoxygenase is involved in LPS-induced pulmonary inflammation**. Neutrophil recruitment into the interstitial (**A**) and BAL (**B**) compartments of the lung and the wet/dry ratio (**C**) of WT mice and *Alox15*^-/- ^mice with (white bars) or without (black bars) LPS inhalation (24 h) was measured by flow cytometry (n = 3 to 4). Exemplary histological images of lungs from WT mice and *Alox15*^-/- ^mice under baseline conditions and after LPS exposure (**D-G**). Bar indicates 50 µm. ^#^*P *< 0.05, *P < 0.05 versus WT LPS.

In order to investigate the role of 12/15-lipoxygenase in pulmonary inflammation, mice were exposed to aerosolized LPS for 30 minutes and neutrophil recruitment into the different compartments of the lung was determined by flow cytometry. In this model, neutrophil recruitment into the lung peaks at 24 h after LPS inhalation [3]. After LPS inhalation, the number of neutrophils transmigrated into the interstitial compartment significantly increased in all mice, but significantly less in *Alox15*^-/- ^mice than in WT mice (Figure 1A). LPS induced an increase of neutrophils in the alveolar space in WT mice, which was reduced by two-thirds in *Alox15^-/- ^*mice (Figure 1B). Elimination of 12/15-LO in *Alox15^-/- ^*mice decreased pulmonary edema formation as measured by wet/dry ratio after LPS exposure (Figure 1C) and decreased histological signs of lung tissue damage (Figure 1D-G). Differential white blood cell counts were not different between WT and *Alox15^-/- ^*mice. All mice survived the 24-hour observation period following LPS administration.

### Pharmacological inhibition of 12/15-LO reduces neutrophil recruitment

**Figure 2:**
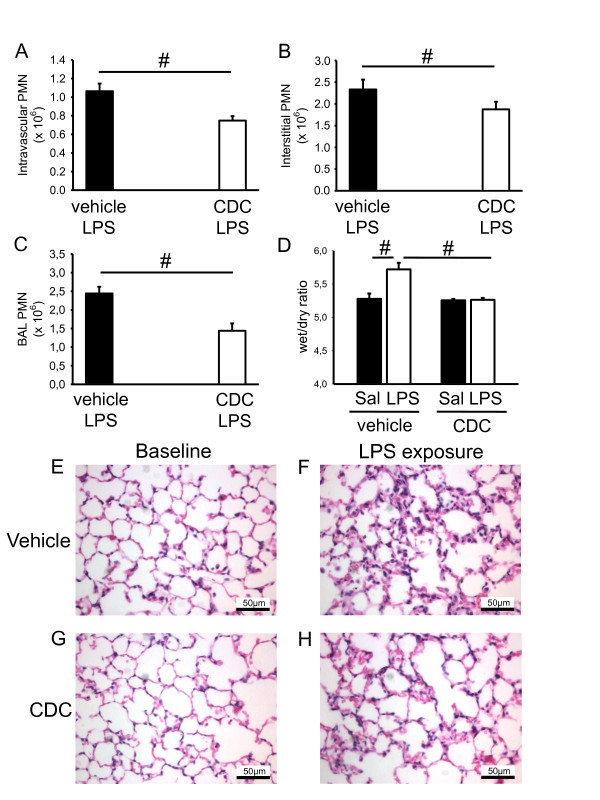
**Pharmacological inhibition of 12/15-LO reduces neutrophil recruitment**. (**A-C**) After pharmacological blockade of 12/15-LO by the inhibitor CDC (Cinnamyl-3,4-Dihydroxy-a-Cyanocinnamate) or in vehicle control mice, neutrophil recruitment (24 h) into the lung was investigated by flow cytometry. Number of neutrophils in the intravascular (A), interstitial (B), and alveolar compartments (C) (n = 4). Wet/dry ratio under baseline conditions and after LPS exposure (**D**) (n = 3). Exemplary histological images of lungs from WT mice and *Alox15*^-/- ^mice under baseline conditions and after LPS exposure (**E-H**). Bar indicates 50 µm. ^#^*P *< 0.05

In order to investigate if the acute blockade of 12/15-LO with a pharmacological inhibitor also reduces neutrophil recruitment, mice were treated with the 12/15-LO inhibitor CDC one hour before LPS exposure. A single injection of 8 mg/kg CDC significantly reduced the 12/15-LO activity, as measured by urinary 12-HETE concentration 24 h later [36]. Pharmacological inhibition of 12/15-LO significantly decreased neutrophil accumulation in the intravascular (Figure 2A), interstitial (Figure 2B) and alveolar compartments (Figure 2C), decreased pulmonary edema formation (Figure 2D) and decreased the histological signs of lung injury (Figure 2E-H) compared to control mice.

### Hematopoietic and non-hematopoietic 12/15-LO is responsible for different steps of neutrophil recruitment into the lung

**Figure 3:**
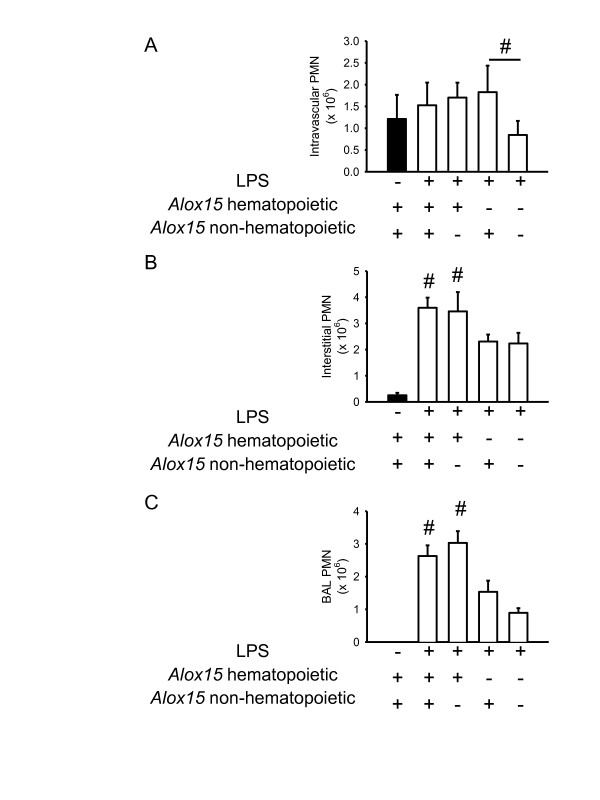
**Hematopoietic 12/15-LO is responsible for the regulation of neutrophil recruitment into the lung**. Neutrophil recruitment into the lung in response to LPS (white bars) was tested in BM chimeras (WT into WT; WT into *Alox15^-/-^*; *Alox15^-/- ^*into WT; *Alox15^-/- ^*into *Alox15^-/-^*) (*n *= 4 to 5 mice per group). Mice lacking hematopoietic and non-hematopoietic *Alox15 *showed a reduced neutrophil accumulation in the intravascular compartment 24 h after LPS inhalation (**A**). Reduced neutrophil recruitment into the interstitial (**B**) and alveolar (**C**) compartment was observed when either hematopoietic or both hematopoietic and non-hematopoietic *Alox15 *was eliminated. n = 4 to 5. ^#^*P *< 0.05.

In order to distinguish between the role of hematopoietic and non-hematopoietic 12/15-LO for neutrophil recruitment, chimeric mice were generated by bone-marrow transplantation. Elimination of either hematopoietic or non-hematopoietic 12/15-LO had no effect on neutrophil accumulation in the intravascular compartment (Figure 3A). Only mice lacking 12/15-LO in both the hematopoietic and non-hematopoietic compartments showed significantly reduced accumulation of neutrophils in the intravascular compartment (Figure 3A), similar to *Alox15*^-/- ^mice (data not shown). The transmigration of neutrophils into the interstitial and alveolar compartment in response to LPS inhalation was reduced in mice lacking 12/15-LO either in the hematopoietic or both compartments (Figure 3B, C).

### Elimination of 12/15-LO activity alters chemokine homeostasis

**Figure 4:**
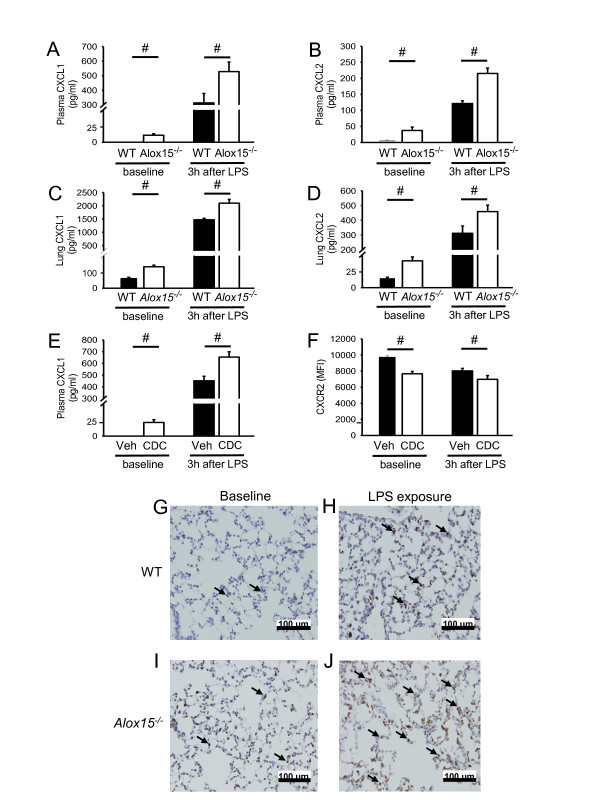
**Elimination of 12/15-LO activity alters chemokine homeostasis**. CXCL1 (**A**) and CXCL2 (**B**) concentrations in the plasma was measured in WT mice (black bars) and *Alox15^-/- ^*mice (white bars) under baseline conditions and 3 h after LPS inhalation (n = 3 to 4). CXCL1 (**C**) and CXCL2 (**D**) concentrations in the lung were measured in WT mice (black bars) and *Alox15^-/- ^*mice (white bars) under baseline conditions and 3 h after LPS inhalation (n = 3 to 4). CXCL1 concentration (**E**) was measured in the plasma of vehicle- or CDC-treated WT mice and CXCR2 expression (**F**) on circulating neutrophils under baseline conditions and 3 h after LPS inhalation was quantified by using flow cytometry (n = 3). (**G-J**) CXCL1 protein expression shown by immunohistochemistry in WT and *Alox15^-/- ^*mice under baseline conditions and 3 h after LPS inhalation. Black arrows indicate CXCL1-producing cells (n = 3). Bar indicates 100 µm. ^#^*P *< 0.05

The chemokines CXCL1 and CXCL2 and their receptor CXCR2 are critically involved in the development of pulmonary inflammation and ALI [5,6,37,38]. Therefore, we measured plasma CXCL1 and CXCL2 concentrations in WT mice and *Alox15^-/- ^*mice under baseline conditions and 3 h after LPS inhalation. *Alox15^-/- ^*mice showed significantly higher CXCL1 and CXCL2 plasma concentrations under baseline conditions compared to WT mice (Figure 4A, B). After LPS exposure, CXCL1 and CXCL2 plasma concentrations were elevated in WT mice and *Alox15^-/- ^*mice and remained higher in *Alox15^-/- ^*mice as in WT mice. Similar to the chemokine concentrations in the plasma, CXCL1 and CXCL2 concentrations in the lung tissue were also higher under baseline conditions and three hours after LPS exposure in *Alox15^-/- ^*mice compared to WT mice (Figure 4C, D). Comparable to genetic ablation of *Alox15*, pharmacological blockade of 12/15-LO in WT mice caused increased CXCL1 plasma levels (Figure 4E) and decreased CXCR2 expression on circulating neutrophils (Figure 4F) compared to vehicle-treated control mice under baseline conditions and after LPS exposure. In order to investigate the location of the CXCL1-producing cells, we conducted immunohistochemistry of the lung for CXCL1. WT mice without LPS inhalation showed almost no CXCL1-producing cells in the lung (Figure 4G), whereas the number of CXCL1-producing cells increased upon LPS exposure (Figure 4H). Lungs from *Alox15^-/- ^*mice showed some CXCL1-producing cells under baseline conditions (Figure 4I) and more after LPS inhalation (Figure 4J).

### Reduced response of neutrophils to CXCL1

**Figure 5:**
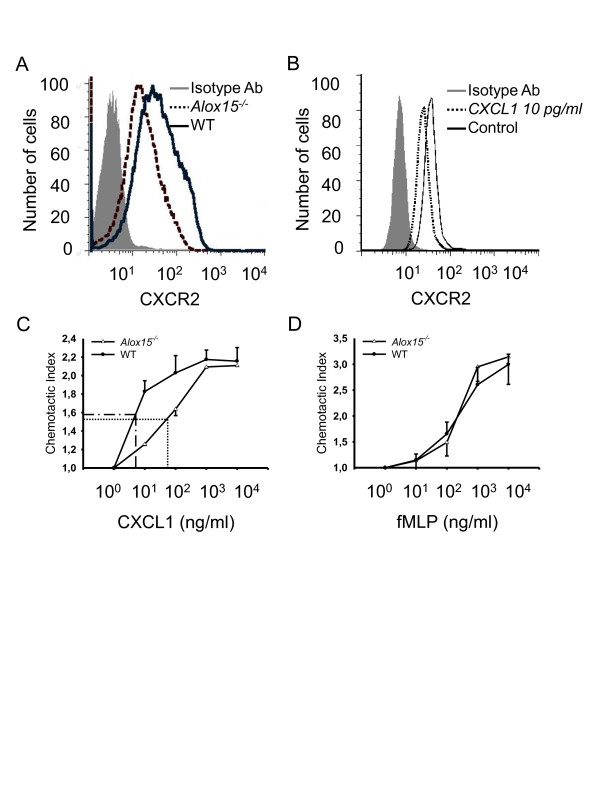
**Elimination of 12/15-LO leads to a reduced response of neutrophils to CXCL1**. (**A**) Flow cytometry analysis of CXCR2 expression was performed on peripheral blood neutrophils from WT and *Alox15^-/- ^*mice under baseline conditions. Whole blood was stained for CD45, 7/4, GR-1 and CXCR2. CXCR2 expression on the surface of peripheral blood neutrophils was determined by using flow cytometry. (n = 3). (**B**) Flow cytometry analysis of CXCR2 expression on isolated neutrophils treated with or without CXCL1 (10 pg/ml) for one hour (n = 6). Migration of labeled neutrophils from WT mice (circles) and *Alox15^-/- ^*mice (triangles) toward different concentrations of CXCL1 (**C**) or fMLP (**D**) was investigated in an *in vitro *chemotaxis assay. Neutrophils were applied on the top of the filter and allowed to migrate toward the chemoattractant for one hour at 37°C. Migrated cells were measured by fluorescence intensity. Chemotactic index was calculated as the ratio of migrated cells with chemoattractant to migrated cells without chemoattractant and ED_50 _was determined (n = 3).

In order to study the consequences of the altered chemokine homeostasis in *Alox15^-/- ^*mice, we investigated CXCR2 expression on neutrophils. The elevated CXCL1 concentration in the plasma of *Alox15^-/- ^*mice led to a significant down-regulation of CXCR2 on the surface of peripheral blood neutrophils from 33 ± 4 to 20 ± 3 mean fluorescence intensity units (n = 3) as measured by flow cytometry (Figure 5A). In order to show that the elevated CXCL1 concentration observed in *Alox15^-/- ^*mice indeed causes the down-regulation of CXCR2 on the neutrophil surface, we incubated isolated neutrophils from WT mice with CXCL1 at a concentration of 10 pg/ml (as measured in the BAL of *Alox15^-/- ^*mice under baseline conditions) at 37°C for one hour and could observe a significantly reduced CXCR2 expression from 35 ± 3 to 18 ± 3 mean fluorescence intensity units (n = 3) in untreated neutrophils (Figure 5B). To test the functional consequences of CXCR2 down-regulation, an *in vitro *chemotaxis assay was performed. Bone marrow-derived neutrophils from both *Alox15^-/- ^*and WT mice showed the same low migration rate without a chemoattractant. Neutrophils from WT mice migrated vigorously towards low concentrations of CXCL1 (10 ng/ml), whereas neutrophils from *Alox15^-/- ^*mice showed a very small response. High CXCL1 concentrations (500 to 10,000 ng/ml) induced similar migration rates in neutrophils from WT and *Alox15^-/- ^*mice. We calculated the chemotactic index (migrated cells with chemoattractant/without chemoattractant) and determined ED_50 _as 4.5 ng/ml in WT mice and 40 ng/ml in *Alox15^-/- ^*mice (Figure 5C). In order to show that the lack or blockade of 12/15-LO specifically abrogates CXCR2-dependent migration, we performed additional chemotaxis assays with the chemoattractant fMLP (n-formyl-methionine-leucine-phenylalanine). There was no difference in the chemotactic index between WT and *Alox15^-/- ^*neutrophils (Figure 5D). Taken together, the increased CXCL1 concentration in the plasma of *Alox15^-/- ^*mice led to reduced response of neutrophils to CXCL1 through a reduced expression of the chemokine-receptor CXCR2 on the cell surface. This and perhaps other desensitization effects led to a reduced neutrophil migration response to physiologically relevant concentrations of CXCL1.

### 12/15-LO regulates neutrophil recruitment in acid-induced acute lung injury

**Figure 6:**
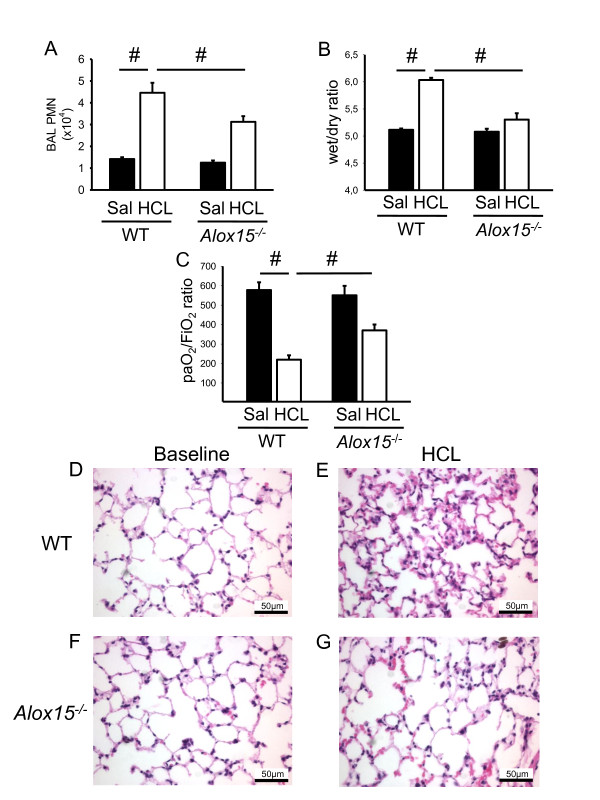
**12/15-LO-deficiency influences acid-induced acute lung injury**. Acid-induced ALI was initiated by intratracheal instillation of 2 µl/g HCL (pH 1.5) in WT and *Alox15^-/- ^*mice. **(A) **After two hours, the lungs were lavaged and the neutrophil count in the BAL fluid was determined (n ≥3 mice). (**B**) Wet/dry ratio of lungs from WT and *Alox15^-/- ^*mice after saline (SAL) or HCL instillation (n = 3). (**C**) Arterial blood was withdrawn from WT mice and *Alox15^-/- ^*mice two hours after saline (SAL) or HCL instillation and the paO_2_/FiO_2 _ratio was calculated (n = 3). Exemplary histological images of lungs from WT mice and *Alox15*^-/- ^mice after saline (SAL) or HCL instillation (**D-G**). Bar indicates 50 µm. ^#^*P *< 0.05

**Figure 7:**
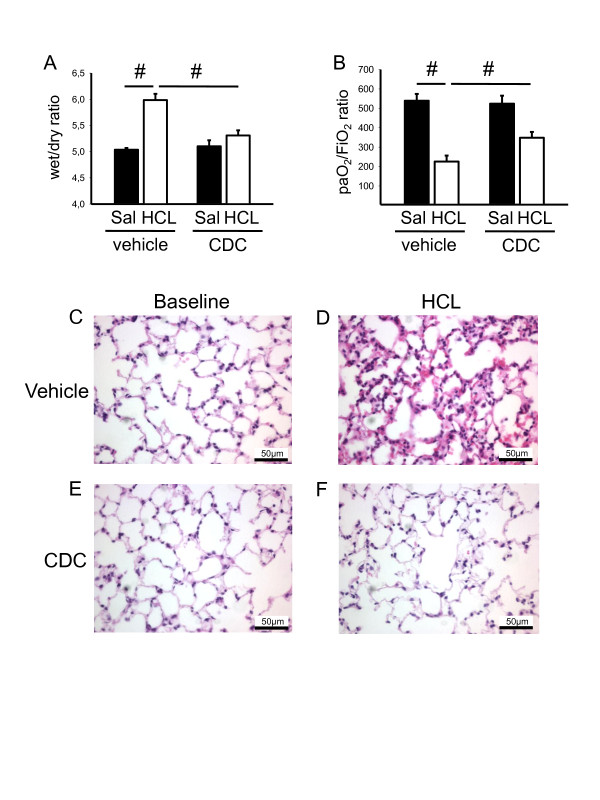
**Pharmacological blockade of 12/15-LO influences acid-induced acute lung injury**. Acid-induced ALI was initiated by intratracheal instillation of 2 µl/g HCL (pH 1.5) in vehicle- and CDC-treated WT mice. (**A**) Wet/dry ratio of lungs from vehicle- and CDC-treated WT mice after saline (SAL) or HCL instillation (n = 3). (**B**) Arterial blood was withdrawn from vehicle- and CDC-treated WT mice two hours after saline (SAL) or HCL instillation and the paO_2_/FiO_2 _ratio was calculated (n = 3). Exemplary histological images of lungs from vehicle- and CDC-treated WT mice after saline (SAL) or HCL instillation (**C-F**). Bar indicates 50 µm. ^#^*P *< 0.05

Based on our finding that the absence of 12/15-LO reduces neutrophil recruitment in response to aerosolized LPS, we hypothesized that this enzyme and its products may also regulate neutrophil recruitment in a clinically relevant model of acid-induced ALI [33]. Two hours after induction of ALI, WT mice showed significantly elevated neutrophil numbers in the BAL (Figure 6A), an increased wet/dry ratio (Figure 6B) and compromised gas exchanges, as measured by the paO_2_/FiO_2 _ratio (Figure 6C), which was significantly attenuated in *Alox15^-/- ^*mice. In addition, the histological hallmarks of acute lung injury, that is, invasion of immune cells, thickening of the interstitial membrane and protein extravasation, were reduced in *Alox15^-/- ^*mice compared to WT animals, suggesting that 12/15-LO regulates neutrophil recruitment and affects edema formation in acid-induced lung injury (Figure 6D-G). Similarly to genetic ablation of *Alox15*, the pharmacological blockade of 12/15-LO decreased the wet/dry ratio (Figure 7A), improved the gas exchange (Figure 7B) and dampened the histological evidence of acute lung injury in tissue samples (Figure 7C-F) in WT mice treated with CDC compared to vehicle-treated control animals.

## Discussion

Our findings suggest that 12/15-LO is critically involved in the regulation of neutrophil recruitment into the different compartments of the lung in LPS-induced pulmonary inflammation and acid-induced ALI. Eliminating or blocking 12/15-LO reduced neutrophil recruitment into the lung in different models of ALI. By using chimeric mice, we demonstrated that hematopoietic and non-hematopoietic 12/15-LO is responsible for different steps of neutrophil recruitment into the lung. Elimination of 12/15-LO reduces neutrophil recruitment into the lung by modulating chemokine/chemokine receptor homeostasis. .

Several studies established the role of 12/15-LO in different chronic inflammatory diseases, including arthritis [39], atherosclerosis [21,40,41] and diabetes [42]. Two products of 12/15-LO, 5-HETE and 12-HETE, have been shown to be implicated in the regulation of vascular permeability during pulmonary inflammation following Gram-negative endotoxemia [43]. A recently published study demonstrated that 12/15-LO-derived 12-HETE regulates vascular permeability in acute lung injury through a CXCR2-dependent mechanism [31]. In previous studies, the elimination or pharmacological blockade of 12/15-LO positively influenced disease outcome, and blocking 12/15-LO has been proposed as a possible anti-inflammatory therapy [44,45]. However, the products of 12/15-LO are different in humans (mainly 15-HETE) and mice (mainly 12-HETE). Here, we show that 12/15-LO regulates neutrophil recruitment into the lung during ALI.

It has been shown that different factors, including different adhesion molecules and chemoattractants, influence neutrophil recruitment into the lung during ALI. It has been demonstrated that the CXCL1-CXCR2 axis is the main factor influencing neutrophil recruitment during ALI [5,7]. Although other chemoattractant factors can influence neutrophil recruitment into the lung [46], a small reduction of CXCR2 on the cell surface may have a substantial impact on neutrophil recruitment [6]. Our data showing that the elimination or pharmacological blockade of 12/15-LO modulates chemokine hemostasis and neutrophil recruitment suggest that mainly the reduced surface expression of CXCR2 is responsible for the diminished severity of ALI. However, we cannot exclude that blocking or eliminating 12/15-LO influences other factors that regulate neutrophil recruitment.

Macrophage-derived 12/15-LO plays an essential role in the development of atherosclerosis in the apoE^-/- ^model [21]. Similar to this study, we show that 12/15-LO expression in hematopoietic cells plays an important role in acute lung injury as measured by interstitial and alveolar neutrophil content. However, non-hematopoietic 12/15-LO is also involved in neutrophil accumulation in the vasculature of the lung. This finding is consistent with the known expression of 12/15-LO in endothelial cells, where the products of the enzyme induce the expression of inflammatory adhesion molecules [26,47]. In the pulmonary vasculature, endothelial cells play an active role in neutrophil recruitment. A recent study has shown that endothelial Gα_i2 _is critical for eosinophil recruitment in ovalbumin-induced allergic airway inflammation [48].

The inhibitory effect on neutrophil recruitment into the different compartments of the lung was stronger in 12/15-LO knockout mice than after pharmacological blockade of 12/15-LO. This effect can be explained by the pharmacokinetics of the inhibitor CDC during the 24 hours between LPS inhalation and mice sacrifice.

The main pro-inflammatory product of 12/15-LO is 12-HETE [43]. Recently, the G protein-coupled receptor GPR31 has been proposed as a receptor for 12-HETE [49]. Knockdown of GPR31 inhibits 12-HETE-induced cell invasion by PC-3M cells [49]. However, it is still unknown whether the stimulation of GPR31 with 12-HETE also regulates vascular permeability, neutrophil recruitment and chemokine release. There is evidence that the stimulation of neutrophils with 12-HETE activates a cascade of downstream molecules, including phosphoinositide 3-kinase, p21-activated kinase (PAK) [50] and p38 mitogen-activated protein kinase [51]. Even though the signaling cascade downstream of 12-HETE is still unknown, the implication of PAK may be a hint for a possible mechanism for the neutrophil effects of 12-HETE. A recent study demonstrated that PAK in neutrophils influences migration into the lung following LPS stimulation [52].

As 12/15-LO produces pro- and anti-inflammatory mediators, the biology of this enzyme is very complex [53]. 12/15-LO is involved in the biosynthesis of 12- and 15-HETE, 13 hydroxyoctadecadienoic acid and lipoxins [54]. 15-HETE and lipoxins have been reported to exert anti-inflammatory activities [55,56]. 15-HETE reduces activation and recruitment of inflammatory cells and modulates the expression of adhesion molecules [57,58]. 12/15-LO is also involved in the production of the anti-inflammatory eicosanoid lipoxin A4 (LXA4). Elimination of 12/15-LO leads to a reduced expression of LXA4 [39]. LXA4 acts by reducing chemokine-sensitivity and consecutively expression of adhesion molecules of effector cells and thus limiting the inflammatory response [59]. These results are well in accordance with our observations that the elimination of 12/15-LO leads to increased CXCL1 levels under baseline and inflammatory conditions. This altered chemokine homeostasis causes the reduced expression of CXCR2 on neutrophils and leads to the diminished neutrophil recruitment into the lung in ALI. We observed a decreased *in vitro *chemotaxis of neutrophils from *Alox15^-/- ^*mice towards lower concentrations of CXCL1 while the chemotactic response was similar at high CXCL1 concentrations. This goes along with the observed CXCL1 concentrations in the lung tissue, which were comparable to the medium range of the concentrations used for the *in vitro *chemotaxis assay.

We confirmed the observations from the model of LPS-induced pulmonary inflammation in a second model of acid-induced lung injury. Although the total numbers of neutrophils recruited to the alveolar compartment 2 hours after acid instillation were lower than 24 hours after LPS inhalation, the recruitment of neutrophils in this model leads to the development of acute lung injury and a compromised gas exchange [33].

## Conclusions

In recent years, the mortality of ALI has been reduced by introducing new ventilation strategies [60], but there are no drugs available to effectively and specifically reduce neutrophil recruitment and vascular permeability to improve survival. The present data suggest that 12/15-LO produced by hematopoietic cells is an important enzyme in regulating the accumulation of neutrophils in the interstitial and alveolar compartments. Therefore, blocking or eliminating 12/15-LO reduces neutrophil recruitment and could lead to a survival benefit in acute lung injury. We could show that inhibition of 12/15-LO by CDC is effective as a pre-treatment. High doses of CDC are required to inhibit 12/15-LO [61-63], thus a more specific agent for the pharmacological blockade of 12/15-LO could be favorable as a possible new therapeutic approach in the treatment of ALI.

## Key messages

• 12/15-LO is required for neutrophil recruitment into the lung during ALI

• 12/15-LO in hematopoietic cells is essential for neutrophil recruitment into the interstitial and alveolar compartment, whereas neutrophil recruitment into the intravascular compartment is regulated by 12/15-LO in hematopoietic and non-hematopoietic cells

• Neutrophil recruitment is disturbed in 12/15-LO-deficient mice, possibly by alterations in the CXCL1/CXCR2 chemokine axis

• Absence of 12/15-LO reduces survival in an acid-induced model of acute lung injury

## Abbreviations

12/15-LO: 12/15-lipoxygenase; 12-HETE: 12-hydroxy-eicosatetraenoic acid; 12-LO: 12-lipoxygenase; 15-HETE: 15-hydroxy-eicosatetraenoic acid; 15-LO: 15-lipoxygenase; 5-HETE: 5-hydroxy-eicosatetraenoic acid; 5-LO: 5-lipoxygenase; ALI: acute lung injury; BAL: bronchoalveolar lavage; CDC: Cinnamyl-3,4-Dihydroxy-a-Cyanocinnamate; CXCL1: keratinocyte-derived chemokine; CXCL2/3: macrophage inflammatory protein 2; CXCR2: C-X-C motif receptor 2; fMLP: n-formyl-methionine-leucine-phenylalanine; HCL: hydrochloric acid; IL-8: interleukin-8; LOX: lipoxygenase; LPS: lipopolysaccharide; LTB4: leukotriene B4; LXA4: lipoxin A4; PAK: p21-activated kinase; PBS: phosphate buffered saline; WT: wild-type.

## Competing interests

The authors declare that they have no competing interests.

## Authors' contributions

JR performed experiments, helped analyze the data and wrote the manuscript. JN and KL helped design the study and revised the manuscript. AZ designed the study, performed experiments, analyzed the results and wrote the manuscript. All authors read and approved the final manuscript for publication.
